# High expression of β3GnT8 is associated with the metastatic potential of human glioma

**DOI:** 10.3892/ijmm.2014.1736

**Published:** 2014-04-08

**Authors:** JUN LIU, LI SHEN, LINGYAN YANG, SHUIJUN HU, LAN XU, SHILIANG WU

**Affiliations:** 1 Department of Biochemistry and Molecular Biology, Soochow University, Suzhou, Jiangsu 215123, P.R. China; 2 Department of Clinical Oncology, Taihe Hospital, Hubei University of Medicine, Shiyan, Hubei 442000, P.R. China; 3 Department of Anatomy, Soochow University, Suzhou, Jiangsu 215123, P.R. China

**Keywords:** β1,3-N-acetylglucosaminyltransferase-8, polylactosamine chains, glioma, invasion and metastasis

## Abstract

Changes in glycosylation due to specific alterations of glycosyltransferase activity have been shown in various tumor cells, including human glioma cells. β1,3-N-acetylglucosaminyltransferase-8 (β3GnT8) catalyzes the formation of polylactosamine on β1–6 branched N-glycans. Upregulated expression of β3GnT8 was described in some tumors, but its precise role in regulating glioma invasion and metastasis remains unclear. In this study, we report on an investigation of the expression of β3GnT8 in human glioma by immunohistochemical analysis. Out of 42 glioma tissues, 37 (88.1%) showed positive β3GnT8 expression, which was significantly higher than that in normal brain tissues (P<0.001). Additionally, the level of β3GnT8 increased with increased pathological grade of gliomas. Silencing of β3GnT8 in U251 glioma cells attenuated the formation of polylactosamine, and decreased cell proliferation, migration and metastatic ability *in vitro* and *in vivo*. By contrast, the overexpression of β3GnT8 in U251 cells exhibited enhanced metastatic potential. A positive correlation between β3GnT8 and matrix metalloproteinase-2 (MMP-2) expression in U251 cells was also observed. The results demonstrated a critical role of β3GnT8 in the metastatic potential of glioma cells, indicating that manipulating β3GnT8 expression may have therapeutic potential for the treatment of malignant glioma.

## Introduction

The carbohydrate moieties of cell surface glycoconjugates participate in processes such as cell-cell, receptor-ligand and glycan-glycan interactions. The biological functions of polylactosamine have been elucidated in several studies. Polylactosamine is a linear carbohydrate polymer composed of repeating N-acetyllactosamine units (Galβ1–4GlcNAcβ1–3)_n_ (1). The glycan chains occur in glycosphingolipids and N-linked/O-linked glycan chains of specific glycoproteins, but are preferentially added to β1–6GlcNAc branches on asparagine-linked oligosaccharides. Polylactosamine on N-glycans is a putative immune regulatory factor presumably suppressing excessive responses during immune reactions (2). Structures of polylactosamine are characteristic of different cell types and stage of differentiation (3). Increased expression of polylactosamine is also associated with tumor cell metastasis and blocking the biosynthesis of these structures inhibits the increased metastatic ability of the cells (4). Some cancer cells, such as U937 cells (histiocytic lymphoma cells), ACHN cells (human kidney glandular cancer cells), MKN45 cells (human gastric cancer cells), A549 cells (human lung cancer cells), and Jurkat cells (acute T-cell leukemia) express a large amount of N-glycans with polylactosamine residues, as analyzed by HPLC and MS techniques (5,6). Comparison of N-glycans from the different colon cancer cells revealed that highly metastatic cancer cells contained more polylactosamine side chains than cells with a low metastatic potential (7).

Polylactosamine is biosynthesized by the alternating action of β1,3-N-acetylglucosaminyltransferase (β3GnT) and β1,4-galactosyltransferase (β4GalT). Both β3GnT and β4GalT are expressed in various human tissues, but their relative expression levels differ between tissues. Therefore, the biosynthesis of carbohydrate structures is tissue-specific and developmentally regulated by glycosyltransferases. There are eight members in the β3GnT family (β3GnT1–T8) and seven members in the β4GalT family (β4GalT1–T7) (8). It has been reported that β3GnT2 and β3GnT8 are mainly responsible for the elongation of polylactosamine. Additionally, the *in vitro* mixing of β3GnT8 and β3Gn-T2 forms a heterocomplex whose enzymatic activity is greatly enhanced, compared with the individual enzymes (9). However, the presence of β3GnT8 can stimulate the activity of β3GnT2. Overexpression of β3GnT8, but not β3GnT2, may induce an increase in polylactosamine chains (10). β3GnT8 was cloned and characterized previously (11,12). Subcellular localization and tumor distribution of β3GnT8 by antiserum showed that the enzyme was expressed significantly higher in some tumor tissues than in normal tissues (13). Moreover, knockdown of β3GnT8 expression by RNAi reduced the tumorigenicity of gastric cancer cells in nude mice (14). To the best of our knowledge, few studies have examined the relationship between the expression of β3GnT8 and metastatic potential in human glioma.

In the present study, the levels of β3GnT8 were measured using immunohistochemical analysis in human glioma tissues. U251 cells were then stably transfected with sense or interference vectors to establish cell lines that overexpressed or were deficient in β3GnT8. We also evaluated the biological function of β3GnT8 in cell invasion and migration *in vitro* and *in vivo*.

## Materials and methods

### Tumor samples

Tissue samples of 42 patients who underwent surgical resections from January, 2003 to December, 2005 were obtained from the First Affiliated Hospital of Soochow University (Suzhou, China). The patients included 23 males and 19 females ranging from 32 to 70 years with a mean age of 49.4 years. Representative tissue blocks from each case were assembled from the archival collections from the Department of Pathology and were graded according to World Health Organization standards, as grade I–II (19 cases), grade III (12 cases), and grade IV (11 cases) tumors. The present study was approved by the Ethics Committees of the First Affiliated Hospital of Soochow University.

### Cell culture and transfection

The U251 human glioma cells were obtained from the American Type Culture Collection (Manassas, VA, USA). The cells were cultured in RPMI-1640 (Invitrogen, Carlsbad, CA, USA) containing 10% fetal bovine serum (FBS). The cells were transfected with pEGFP-C1, pEGFP-C1-β3GnT8, or pSilencircle-β3GnT8Scr, pSilencircle-β3GnT8Si plasmids by Lipofectamine 2000 (Invitrogen) as described previously (15). Stable polyclonal cells were established under continuous selection with geneticin (G418; Invitrogen). The stable cells were correspondingly designated as Mock vs. T8S, and T8Scr vs. T8Si. Untransfected cells were indicated as NC.

### RNA extraction and reverse transcription-polymerase chain reaction (RT-PCR) assay

Total RNA was extracted from glioma cells by TRIzol (Invitrogen). RNA (1 μg) was used as a template for cDNA synthesis, which was performed using First-Strand Synthesis system for RT-PCR kit (Invitrogen). RT-PCR analysis was performed using the primer: human β3GnT8 (sense, 5′-CCCTGACTTCGCCTCCTAC-3′ and antisense, 5′-GGTCTTTGAGCGTTCGGTTGA-3′, product size, 362 bp); matrix metalloproteinase-2 (MMP-2) (sense, 5′-AAC CCTCAGAGCCACCCCTA-3′ and antisense, 5′-GTGCATACA AAGCAAACTGC-3′, product size, 285 bp); tissue inhibitors of metalloproteinase-2 (TIMP-2) (sense, 5′-AAACGACAT TTATGGCAACCC-3′ and antisense, 5′-ACCCAGTCCATC CAGAGGC-3′, product size, 359 bp); β-actin (sense, 5′-CCTCTATGCCAACCACAGTGC-3′ and antisense, 5′-GTA CTCCTGCTTGCTGATCC-3′, product size, 250 bp). β-actin was used as a housekeeping gene control. Amplification reaction protocol was performed for 30 cycles at 94°C for 40 sec, 52°C for 30 sec, and 72°C for 40 sec. Products were analyzed by 1.5% agarose gel electrophoresis and visualized by ethidium bromide staining.

### Antibodies and western blot analysis

Rabbit anti-human β3GnT8 affinity pAb was purified by our Laboratory (13). Anti-MMP-2 and anti-TIMP-2 monoclonal antibody, anti-β-actin antibody, anti-rabbit-HRP secondary antibody and anti-mouse-HRP secondary antibody were purchased from Santa Cruz Biotechnology, Inc. (Santa Cruz, CA, USA). Western blot assay was conducted with the standard methods. Briefly, equal amounts of protein from total cell lysates were separated on 10% SDS-PAGE and transferred to polyvinylidene difluoride membrane. After blocking with 5% fat-free milk in phosphate-buffered saline (PBS) for 2 h at room temperature, the membranes were incubated with the primary antibody overnight at 4°C followed by incubation with secondary antibody. The proteins were visualized using an ECL detection kit purchased from Beyotime Institute of Biotechnology (Nantong, China). Relative optical densities of protein bands to that of loading control (β-actin) were quantified by Scion Image software.

### Flow cytometric analysis of cellular glycosylation

Cells were washed, collected from plates, and centrifuged at 1,500 × g for 3 min. The precipitate was resuspended in 100 μl of PBS. The cells were incubated at 37°C with 0.5 μg/ml biotin-conjugated *Lycopersicon esculentum* agglutinin (tomato lectin, LEL; Sigma, St. Louis, MO, USA). After 1 h, the cells were washed and bound lectin was detected with phycoerythrin-conjugated streptavidin (Sigma) for 30 min at 37°C. Cell samples were subjected to flow cytometry with unstained cells serving as the control. Fluorescence histograms and mean fluorescence data were created and analyzed with CellQuest software.

### MTT assay

MTT assay was used to assess the effect of β3GnT8 on glioma cell proliferation. Different group cells were plated at a density of 5×10^3^/well in 96-well plates and incubated for 24, 48, 72, 96 and 120 h under complete culture medium. MTT (Sigma) was dissolved in PBS at 5 mg/ml and filtered to be sterilized, and 20 μl MTT solution was added at different time-points. Plates were then incubated at 37°C for 4 h, 100 μl dimethylsulfoxide was added to each well and mixed thoroughly to dissolve the blue-violet crystals. Cell viability data were measured with an ELISA reader at 490 nm.

### Colony formation assay

Cells were plated in 6-well plates (5×10^3^ cells/well) and cultured in medium with 10% FBS and G418 (500 μg/ml). The cells were then incubated for 21 days until colonies were large enough to be visualized. The colonies were stained with 0.5% crystal violet for 30 min after fixation with methanol for 30 min at room temperature.

### Transwell assay

The invasion assay was performed in 24-well cell culture chambers using Transwell inserts (Corning Life Sciences, Corning, NY, USA) with 8 μm Pore membrane precotated with Matrigel (BD Bioscienses, Franklin Lakes, NJ, USA). Cells (1×10^5^) were plated in the upper compartment in 200 μl serum-free medium per chamber, and 500 μl of complete serum medium was added to the lower wells. The cells were allowed to invade for 24 h, after which, the non-invading cells with Matrigel matrix were removed from the upper surface of the membrane by scrubbing with a cotton-tipped swab. The cells on the lower surface of the filter were fixed for 30 min in 4% polyoxymethylene, air-dried briefly, and stained with crystal violet (0.1%). The number of invaded cells was counted from 15 randomly selected microscopic fields at a magnification of ×200.

### Wound-healing assay

Cells were plated in a 6-well plate at equal numbers of 1×10^5^ and incubated overnight, yielding confluent monolayers. Wounds were made using a pipette tip and images were captured immediately (time zero) and 24 h after wounding. The area migrated by the cell monolayer to close the injury line was measured. The plates were marked to ensure consistent photodocumentation. Using the ImageJ software, the area of each wound was calculated at each time-point.

### Tumor growth in nude mice

Four-week-old female nude mice (SPF BALB/c, SCXK 2007-0005) obtained from the Laboratory Animal Center of Soochow University were used for the *in vivo* studies. Each experimental group consisted of four nude mice. After being grown to subconfluency, transfected (pEGFP-C1-β3GnT8 and pSilencircle-β3GnT8Si) and non-transfected cells were harvested by trypsinization, centrifuged, resuspended in 0.2 ml PBS at a density of 1×10^7^ cells/0.2 ml, and injected into nude mice. Tumor size was calculated using the formula T_vol_ = width^2^ × length × 0.5. The growth curve of each tumor was plotted. After four weeks, mice were sacrificed and tumors were removed and excised. The animal studies were approved and supervised by the Research Ethics Committee of Soochow University.

### Immunohistochemistry

For immunohistochemical staining, the clinical samples were fixed in freshly prepared 10% neutral-buffered formalin, embedded in paraffin, and cut into 4 μm sections. After baking at 60°C overnight, the sections were dewaxed and rehydrated. Endogenous peroxidase activity was blocked by incubation in 3% hydrogen peroxide for 10 min at room temperature. After washing with PBS, the sections were incubated with β3GnT8 pAb at 4°C overnight. HRP-labeled secondary antibody using the MaxVision™ HRP-Polymer anti-mouse/rabbit IHC kit (Maixin Bio, Fuzhou, China) was applied and incubated for 30 min at room temperature, followed by 5-min incubation at room temperature with DAB provided in the kit for color development. The negative control was treated with PBS instead of β3GnT8 pAb.

One thousand cells of each section were randomly selected and counted blindly by three independent observers. The degree of immunostaining of the sections was viewed and scored separately by two independent investigators. The scores were determined by combining the proportion of positively stained tumor cells and the intensity of staining. Scores from the two investigators were averaged for further comparative evaluation of the β3GnT8 expression. The proportion of positively stained tumor cells was graded as (16): 0 (<5% positive tumor cells), 1 (<6–25% positive tumor cells), 2 (26–50% positive tumor cells) and 3 (>50% positive tumor cells). The intensity of staining was recorded on a scale of 0 (no staining), 1 (weak staining, light yellow), 2 (moderate staining, yellowish brown) and 3 (strong staining, brown). The scores for the percentage of positive tumor cells and for the staining intensity were added to generate an immunoreactive score for each specimen. The product of the quantity and intensity scores were calculated such that a final score of 0 indicated a negative expression (−), 1–4 indicated a weak expression (+), 5–8 indicated a moderate expression (++) and 9–12 indicated a strong expression (+++).

### Statistical analysis

SPSS statistical software for Windows 13.0 (SPSS, Inc., Chicago, IL, USA) was used for all statistical analyses. Differences of tumor cell proliferation and invasion between the groups were analyzed by the t-test. The Jonckheere-Terpstra test was used to correlate cumulative β3GnT8 expression with glioma grading, and the Chi-square test was used for comparisons between groups. All the experiments were repeated at least three times. The results were presented as means ± standard deviation (SD). P<0.05 was considered to indicate statistical significance.

## Results

### Correlation between the expression of β3GnT8 and malignancy of gliomas

**Figure 1:**
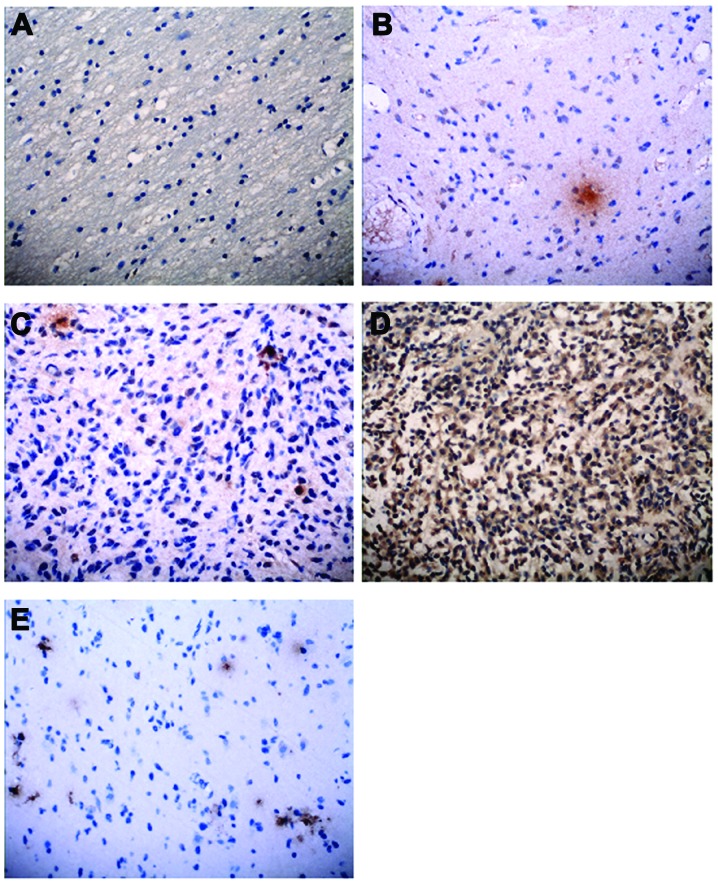
Immunohistochemical analysis of β1,3-N-acetylglucosaminyltransferase-8 (β3GnT8) in glioma tissues and normal brain tissues. The figures showed the strongly cytoplasmic staining of β3GnT8 in glioma tissues. (A) Normal brain tissue; (B) WHO grade II; (C) WHO grade III; (D) WHO grade IV; (E) negative control (magnification, ×200).

To determine whether the expression level of β3GnT8 protein is associated with the histological characteristics of glioma tissues, immunohistochemistical analysis of human glioma tissues and normal brain tissues was performed with β3GnT8 pAb. In the present study, during immunohistochemical staining, primary antibody control was replaced by normal rabbit serum, which was widely used as a negative control in the immunoassay. β3GnT8 exhibited strong cytoplasmic staining (brown nuclei) in cancer cells (Fig. 1). Out of the 42 glioma tissues, 37 (88.1%) were positive for β3GnT8 expression, whereas only 12.5% (1/8) of normal brain tissues showed β3GnT8 staining. The difference of β3GnT8 expression between the glioma tissues and normal brain tissues was significant (P<0.01). The correlation between the expression of β3GnT8 protein and the histological staging of gliomas was also analyzed. As shown in Table I, β3GnT8 expression was significantly higher in high-grade gliomas (grade III and grade IV) compared to low-grade gliomas (grade I–II) (P<0.05), confirming that β3GnT8 expression is associated with the progression of glioma. On the other hand, the relationship between intensity of β3GnT8 and factors such as age and gender was not observed (P>0.05).

### Establishment of β3GnT8 overexpression or knockdown cell lines

**Figure 2:**
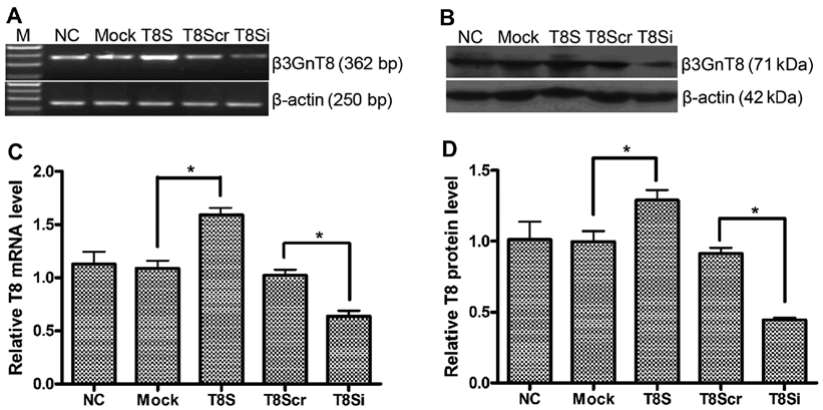
Expression of β1,3-N-acetylglucosaminyltransferase-8 (β3GnT8) mRNA and protein in different groups. (A) Reverse transcription-polymerase chain reaction (RT-PCR) results of β3GnT8 mRNA. (B) Western blot results of β3GnT8 protein. (C) Analysis of β3GnT8 relative mRNA level. (D) Analysis of β3GnT8 relative protein level. Data were obtained from triplicate experiments and are indicated as means ± standard deviation (SD). ^*^P<0.05.

To investigate the potential role of β3GnT8 in glioma, β3GnT8 overexpressing or knockdown cells were established using U251 cells as the parental cell line. Stable cell clones were selected with selection medium containing 500 μg/ml G418 and confirmed by RT-PCR and western blotting. β3GnT8 expression at mRNA and protein levels was significantly increased in the T8S group in comparison to the T8Si group (P<0.05) (Fig. 2). No significant difference was observed in β3GnT8 expression between the NC, Mock and T8Scr groups (P>0.05). Thus, these stable cell lines were effectively used for subsequent experiments.

### Alteration of polylactosamine chains affected by β3GnT8 expression

**Figure 3:**
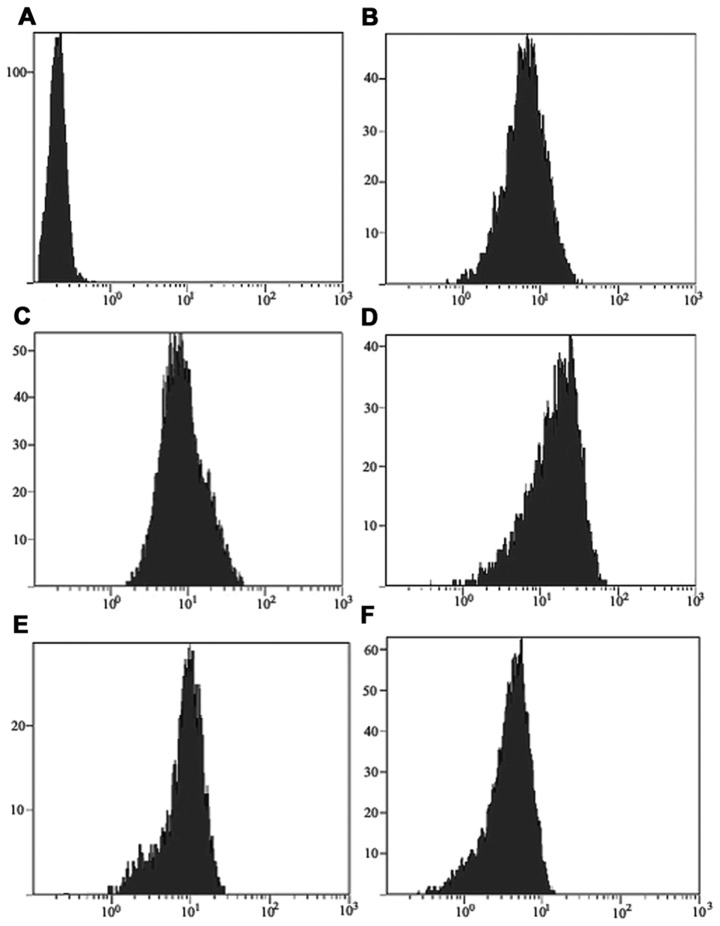
Analysis of polylactosamine chains by flow cytometry. The expression of polylactosamine was recognized by LEL, which was replaced by phosphate-buffered saline (PBS) and used as a control. The intensity of LEL staining was shown as the X-Mean. In the groups (A) control; (B) NC; (C) Mock; (D) T8S; (E) T8Scr; and (F) T8Si, the X-Mean was 0.03±0.02, 7.08±0.14, 7.57±0.05, 17.61±0.21, 7.83±0.17 and 4.45±0.09, respectively.

We examined whether the oligosaccharide structures of N-glycans were associated with altered β3GnT8 expression. U251 cells stained with LEL were subjected to flow cytometry analysis. As this lectin recognizes ≥3 linear units of repeating lactosamine, it is suitable for detection of relatively long polylactosamine chains (10). LEL was replaced by PBS, which was widely used as the control in the flow cytometric analysis of cellular glycosylation. The formation of polylactosamine chains was increased in T8S cells, but decreased in T8Si cells (P<0.05) (Fig. 3). The intensity of LEL staining was shown as the X-Mean. In the control, NC, Mock, T8S, T8Scr, T8Si groups, the X-Mean was 0.03±0.02, 7.08±0.14, 7.57±0.05, 17.61±0.21, 7.83±0.17 and 4.45±0.09, respectively. This result confirmed that β3GnT8 is involved in the biosynthesis of polylactosamine chains.

### Effect of β3GnT8 on cell proliferation and growth in vitro and in vivo

**Figure 4:**
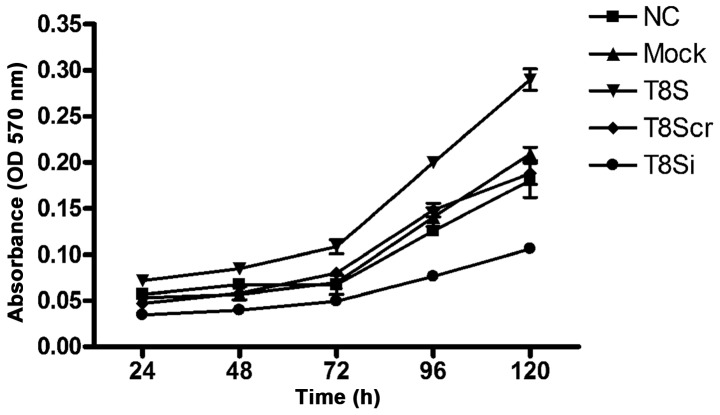
Effect of β1,3-N-acetylglucosaminyltransferase-8 (β3GnT8) on the proliferation of U251 cells *in vitro*. Cell proliferation was investigated by MTT assay at different time-points after culture at 24, 48, 72, 96 and 120 h.

The MTT assay was performed to investigate the effect of β3GnT8 on glioma cell proliferation. Our results showed that β3GnT8 did not influence the cell proliferation within 48 h (Fig. 4). With the longer time, upregulation of β3GnT8 resulted in significant promotions of cell proliferation compared with cells in NC, Mock and T8Scr groups (P<0.05). Proliferation of the glioma cells in T8Si group at same time-points was inhibited significantly compared with that of NC, Mock and T8Scr (P<0.05).

**Figure 5:**
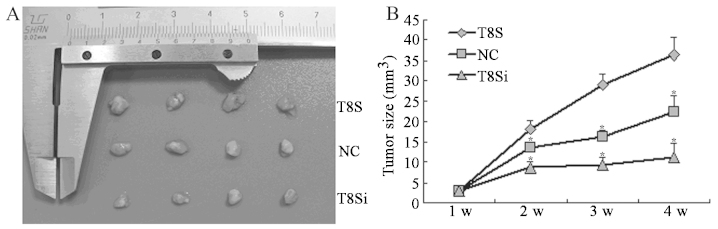
Tumor growth in the nude mice injected with T8S, T8Si or non-transfected cells within four weeks post-injection. (A) Representative image of tumor. (B) The tumor growth curve showed a significant growth tendency in mice injected with T8S cells (^*^P<0.05 compared to the NC and T8si group).

Furthermore, transfected (T8S and T8Si) and non-transfected (NC) cells were inoculated into nude mice to examine the effect of β3GnT8 on xenograft tumor formation. Tumor growth *in vivo* was promoted in T8S group but inhibited in T8Si group (Fig. 5). The average tumor size was smaller in mice injected with β3GnT8 knockdown cells (11.211±3.569 mm^3^) than non-transfected (22.383±3.821 mm^3^; P<0.05) and β3GnT8 overexpression cells (36.350±4.424 mm^3^; P<0.05). These data indicate that expression of β3GnT8 affects tumorigenicity of glioma cells.

### Effect of β3GnT8 on metastatic ability in vitro

**Figure 6:**
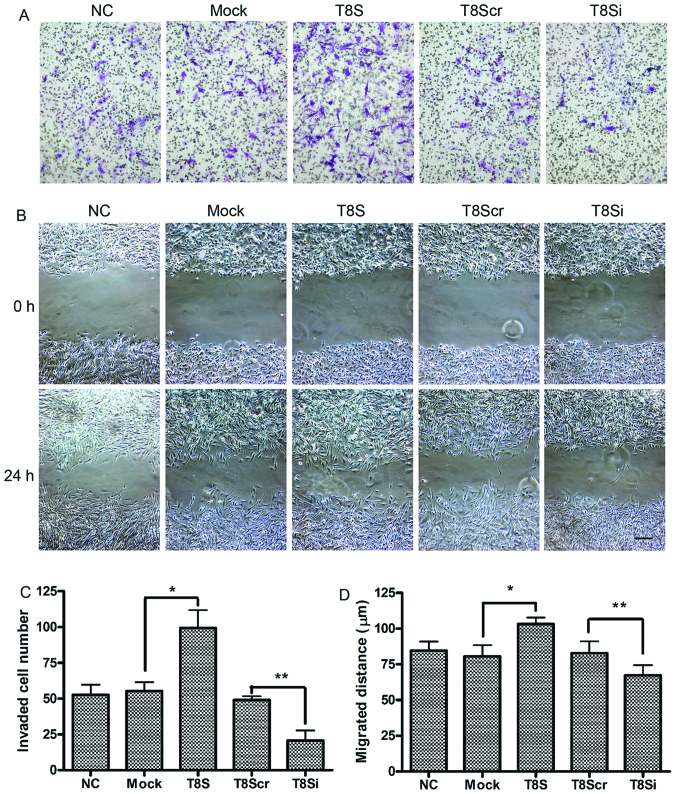
Cell metastatic capability detection. (A) Represent photomicrographs showing invasive capability of U251 cells in a chemoinvasion chamber (×200 magnification). (B) An injury line was made on a confluent monolayer of cells. Cell motility was examined with a light microscope (magnification, ×40) at the indicated time-points (0 and 24 h). (C) The number of migrating cells was determined by counting the stained cells and histogram. (D) The wounding area was quantified. ^*^P<0.05 compared to the Mock group; ^**^P<0.05 compared to the T8Scr group.

**Figure 7:**
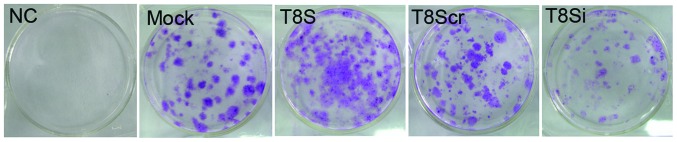
Colony-formation assay was performed in 6 cm dishes and images of cell colonies were captured (magnification, ×40).

To assess the effect of β3GnT8 stimulation on the invasion behavior of glioma cells, we performed a Transwell assay. β3GnT8 overexpression in the T8S group exhibited a greater potential to migrate than NC, Mock and T8Scr cells (Fig. 6A and C). By contrast, the migration of U251 cells with silenced β3GnT8 expression was impeded compared to that of NC, Mock and T8Scr. Similarly, in the wound-healing assay, within 24 h after scratching, the U251 cells in the T8S group were more completely healed than T8Si cells (Fig. 6B and D). Soft-agar colony-formation assay *in vitro* confirmed the effect of β3GnT8 on tumor metastatic capability. The colony numbers and size of U251 cells in the T8Si group were apparently less and smaller than those of the Mock and T8Scr cells, whereas more and larger colonies formed in the β3GnT8 overexpressed group (Fig. 7). However, single cell clones in the NC group were not found as G418 was added to the culture medium. Thus, the results suggested that β3GnT8 expression markedly affects cell migration and metastatic ability.

### Effect of β3GnT8 on the expression of MMP-2 and TIMP-2

**Figure 8:**
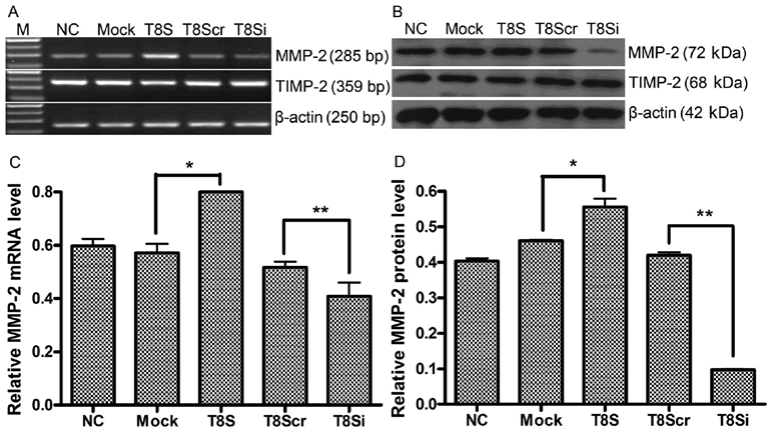
The effects of β1,3-N-acetylglucosaminyltransferase-8 (β3GnT8) on expression of matrix metalloproteinase-2 (MMP-2) and tissue inhibitors of metalloproteinase-2 (TIMP-2) at mRNA and protein levels. (A) The mRNA expression levels of MMP-2, TIMP-2 or β-actin (as an internal control) were determined by reverse transcription-polymerase chain reaction (RT-PCR). (B) The expression levels of MMP-2, TIMP-2 or β-actin (as an internal control) were determined by western blotting. (C and D) mRNA and protein expression levels of MMP-2 were quantified after normalization to β-actin. Results are indicated as mean ± standard deviation (SD). ^*^P<0.05 compared to the Mock group; ^**^P<0.05 compared to the T8Scr group.

Degradation of the ECM is an important process associated with tumor invasion, in which MMP-2 play a critical role. TIMP-2, as a natural inhibitor of MMP-2, promotes the effective activation of proMMP-2 (17). We examined whether β3GnT8 affected the expression of MMP-2 and TIMP-2 by RT-PCR and western blotting. We found that overexpression of β3GnT8 in U251 cells induced an increase in the levels of MMP-2 compared with NC, Mock and T8Scr cells (P<0.05) (Fig. 8). Furthermore, a significant reduction of MMP-2 levels was detected following knockdown of β3GnT8 in U251 cells (P<0.05). However, the expression of TIMP-2 exhibited no obvious changes in the β3GnT8 overexpression or knockdown cells. These findings suggested that the effects of β3GnT8 on migration and metastatic of U251 may occur primarily by affecting the expression of MMP-2.

## Discussion

Glioma is the most frequent tumor originating from glial cells in the nervous system, and is characterized by rapid cell proliferation and a high level of invasiveness in the surrounding brain (18). High-grade gliomas are the most common human brain tumors and are essentially incurable. Therefore, it is essential to investigate the mechanisms underlying the development and progression of gliomas. The experiments presented in this study are predicated on the hypothesis that alterations in the expression of cell surface carbohydrates modulate the invasive potential of malignant gliomas. To the best of our knowledge, this is the first study to demonstrate that β3GnT8 expression increased significantly from low-grade (grade I–II) to high-grade (grade III and IV) glioma. Other biological factors such as age and gender were not associated with β3GnT8 expression. The results of the present study also showed that the level of β3GnT8 correlated positively with the metastatic potential of glioma cells.

Aberrant glycosylation of cell-surface glycoconjugates is a universal feature of cancer cells. These alterations may be instrumental in the failure of intercellular contact and communication and in the invasive and infiltrative properties of cancer cells. Correlations of disease phenotypes with glycosylation changes have been analysed intensively in the tumor biology field. Additionally, numerous studies (19–23) have focused on the effect of aberrant sugar chains in malignant phenotype of human glioma cells. For example, glioma cells express extremely high levels of cell-surface α2,3-linked terminal sialic acids on glycoproteins bearing N-linked glycans (19). The linkage and expression levels of the terminal sialic acids play an important role in glioma cell-extracellular matrix (ECM) interactions. Transfection of α2,6- and α2,3-sialyltransferase genes and GlcNAc transferase genes into U-373 MG human glioma cells affects glycoconjugate expression and enhances cell death (20). Polysialic acid is a large glycan with restricted expression, typically found to be attached to the protein scaffold neural cell adhesion molecule (NCAM). Polysialylated NCAM, which is found in various types of cancer, including gliomas, is positively associated with metastasis and disease progression (21). β4GalT5 could effectively galactosylate the GlcNAcβ1–6 branch which is a marker of glioma. It has been reported that the expression of β4GalT5 is increased in the process of glioma development (22). β4GalT5 regulates the self-renewal of glioma-initiation cells and may be a novel target in glioma (23). Downregulation of β4GalT5 may promote the expression of cell surface integrin β1 and subsequently inhibit glioma malignant phenotype, and may enhance the therapeutic efficiency of As_2_O_3_ (24,25). Findings of our previous study showed that ppGalNAc-T2, which catalyzes initiation of mucin-type O-glycosylation, regulated the invasion and migration of human glioma cells *in vitro* (26). However, the roles of β3GnT8 in tumor metastatic potential have not been adequately studied. Thus, the present study focused on the regulation of β3GnT8 expression during oncogenesis and metastasis in human glioma cells.

To investigate the role of β3GnT8 on the malignant phenotype of glioma, exogenous β3GnT8 was introduced into U251 cells, and the expression of β3GnT8 was downregulated in U251 cells. We also investigated whether polylactosamine chains in the cell surface of the tumor were altered by β3GnT8. LEL was initially used to detect cancer-specific changes of glycosylation on N-glycans. Lectins are carbohydrate-binding proteins or glycoproteins of non-immune origin that recognize and reversibly bind to glycans without altering their covalent structure. Our results indicate that engineered overexpression of β3GnT8 in U251 cells increased the polylactosamine chains, leading to an increase in the migration and metastatic ability of glioma cells. Conversely, downregulated expression of β3GnT8 in U251 cells showed reduced polylactosamine, and significantly decreased the migration and metastatic ability. β3GnT8, which is involved in the formation of glycans, plays an important role in tumor progression and metastasis.

Metastasis is the major cause of death among glioma patients. Metastasis of glioma cells requires degradation of ECM, thereby modifying the extracellular environment by overexpression of proteolytic enzyme activity, such as MMPs. MMPs are a growing family of zinc-dependent endopeptidases belonging to the M10A subfamily, which are capable of degrading various components of the ECM including collagens, laminin, fibronectin, vitronectin and proteoglycans. A growing body of evidence suggests that MMPs, particularly MMP-2, is upregulated in malignant tumors and contributes to the invasion and metastatic spread of cancer cells by degrading type IV collagen, a major component of the basement membrane (27,28). MMP-2 is highly expressed in gliomas as compared to normal brain tissue. MMP-2 activates several key molecules leading to rapid cell proliferation, increased motility, invasion, and angiogenesis of gliomas. Adenovirus-mediated transfer of siRNA against MMP-2 mRNA results in impaired invasion and tumor-induced angiogenesis, induces apoptosis *in vitro* and inhibits tumor growth *in vivo* in glioblastoma (29). However, the regulation of MMP-2 expression is complex. It is noteworthy in this study that the overexpression of β3GnT8 led to the increase of MMP-2 in U251 cells. By contrast, a decreased MMP-2 expression was observed in β3GnT8 silenced U251 cells. We also investigated the effect of β3GnT8 on the expression of tissue TIMP-2, a natural inhibitor of MMP-2. It appeared that TIMP-2 did not interact with β3GnT8. However, there is no clear experimental evidence of direct interactions between MMP-2 and β3GnT8.

Most of the proteins in the organism are glycoproteins. Although in the present study, MMP-2 is a non-glycosylated protein, it is now appreciated that the expression of MMP-2 can be induced by glycoproteins containing polylactosamine chains. For example, CD147 is enriched on the surface of many malignant tumor cells. As a result of heterogeneous N-glycosylation, CD147 exists in a highly glycosylated form, HG-CD147 and a lowly glycosylated form, LG-CD147. HG-CD147 contains N-acetylglucosaminyltransferase V-catalyzed, β1, 6-branched, polylactosamine-type sugars, which account for its excess size (30). As a transmembrane glycoprotein, HG-CD147 forms homo-oligomers in the heterotypic and homotypic cell-cell interactions to induce the production of MMP-2 (31). In addition, galectin-3 also expresses polylactosamine-type N-glycans in cancer cells (6). Galectin-3 may be used as a simple, rapid, and reliable surrogate marker for the activities of MMP-2 (32). Since glycoproteins are mainly distributed on the cell surface, and are involved in numerous biological behaviors of cell, such as signal transduction, we hypothesized that β3GnT8 affects cellular signal transduction by altering the structure of glycans on the glycoproteins, and also influences the expression of MMP-2. However, these aspects remain to be investigated.

In conclusion, we have shown that the level of β3GnT8 significantly increases with an increased pathological grade of gliomas. After stable transfection of U251 cells with exogenous β3GnT8 cDNA, a marked improvement on the migration and metastastic potential was observed. On the other hand, the inhibition of migration and metastasis was associated with the downregulated β3GnT8 expression in U251 cells. Our studies suggest that high levels of β3GnT8 are associated with the metastatic potential of cancer cells. This property may provide useful information for the diagnosis and prognosis of glioma.

## Figures and Tables

**Table I:** β3Gn-T8 expression is correlated with glioma progression.

		Degree of β3Gn-T8 immunoreactivity				
			
Variables	Case (n)	−	+	++	+++	P-value
Age						>0.05
≥50	23	4	6	6	7	
<50	19	1	6	6	6	
Gender						>0.05
Female	19	2	5	5	7	
Male	23	3	7	7	6	
Characteristics						<0.05
I–II	19	4	9	5	1	
III	12	1	2	4	5	
IV	11	0	1	3	7	

β3Gn-T8, β1,3-N-acetylglucosaminyltransferase-8.
